# The effectiveness of different immunotherapies in the treatment of condyloma acuminatum: a network meta-analysis of randomized clinical trials

**DOI:** 10.3389/fmed.2023.1260139

**Published:** 2023-11-21

**Authors:** Xiaoye Liu, Manli Qi

**Affiliations:** Department of Dermatology, Tianjin Union Medical Center, Tianjin, China

**Keywords:** acuminatum, HPV, immunotherapy, BCG, MRR, Candida antigen, PPD, vitamin D3

## Abstract

**Background:**

The treatment of condyloma acuminatum (CA), especially the very persistent and recurrent CA, is currently the focus of our research. Immunotherapies have recently been shown to be well-tolerated and effective in treating warts, particularly refractory warts. However, there is still a lack of corresponding evidence-based medical evidence on the effectiveness of different immunotherapies in treating warts. The difference between network meta-analysis and meta-analysis is that network meta-analysis can be used to compare multiple treatments by combining direct and indirect evidence to assess the interrelationship between all treatments. We intend to compare the efficacy of different treatments for CA using a network meta-analysis.

**Methods:**

PubMed, Cochrane Library and Embase from inception to June 1st, 2023 were searched using a computer. All articles on immunotherapies for CA were included. Stata MP17.0 software was used for data analyses.

**Results:**

A total of 8 randomly-controlled trials involving 493 patients were included. Result showed that all treatment measures had a significant efficacy compared with the regular saline group (BCG (bacillus Calmette-Guérin vaccine) OR = 96.00, 95%CI: 10.35–890.58; MMR (measle, mumps and rubella vaccine) OR = 29.69, 95%CI: 7.47–118.04; Candida antigens OR = 27.34, 95%CI: 8.64–86.52; PPDs (purified protein derivatives) OR = 23.33, 95%CI: 6.75–80.60; VD3 OR = 21.36, 95%CI: 4.34–105.16 and purified protein derivatives (general) OR = 13.14, 95%CI: 3.38–51.12). The area under the curve (SUCRA) ranking results showed that the bacillus Calmette-Guérin vaccine had the highest total efficiency, which was 88.2%, with the rest in the order of measle, mumps and rubella vaccine, which was 68.9%, Candida antigens, which was 63.6%, purified protein derivatives, which was 52.9%, vitamin D3, which was 49.0%, purified protein derivatives (general), which was 27.4%, and saline, which was 0%.

**Conclusion:**

In summary, we found that the bacillus Calmette-Guérin vaccine was superior to other treatments in terms of efficacy according to the SUCRA value.

## 1 Introduction

Condyloma acuminatum (CA) is a cauliflower-shaped wart that appears on the genital area due to human papillomavirus (HPV) infection, which is most common among young people and is more common among women than men (1). Sexual transmission is the primary transmission mode of CA; the incubation period varies from 1 to 12 months, with an average of 2–3 months. There are also cases of auto-inoculation and vertical transmission. 90% of CA is caused by 2 low-risk subtypes, 6 and 11 (2), while some are also associated with other subtypes, such as 16, 18, 31, 33, and 35. Due to the moist environment of the genital area and excessive folds in the cavity, viruses have an easy chance to hide with a low spontaneous clearance by the body; warts are widely spread, which have a high recurrence rate. Some high-risk types, such as 16 and 18, may also lead to the development of cervical cancer, which imposes a specific psychological burden on the population. Currently, the treatment of CA, incredibly stubborn CA, is the focus of our research.

Traditional treatment modalities include chemical cautery, electrocautery, cryotherapy, surgical excision and laser removal, which can cause various adverse effects, such as pains, disfigurement and infections. In contrast, through immunotherapies, warts can be removed from the skin surface and the viruses can be eliminated by enhancing cell-mediated immunity. Recently, the intra-focal immunotherapy is a new treatment modality, such as measle, mumps and rubella (MMR) vaccines, purified protein derivatives (PPDs), the bacillus Calmette-Guerin (BCG) vaccines and the Candida antigens, which are well-tolerated and effective in the treatment of CA, particularly stubborn CA. MMR and PPDs have been reported to be most effective in achieving a complete clearance and long-term maintenance with reduced recurrence rates at the same site compared to other treatment modalities (3).

The exact mechanism of the intralesional immunotherapy has yet to be entirely understood, through which cell-mediated immunity to the HPV virus can be enhanced. The preliminary view is that the induction of a delayed hypersensitivity is the primary mechanism. Using immunotherapies, immediate cellular immunity can be increased by inducing a systemic T-cell-mediated response that increases the release of Th1 cytokines (IL-1 and IFN-γ) and the downregulation of Th2 cytokines (IL-10), leading to the clearance of warts (4). Hadeer et al. (5) found an elevated IL-18 level after PPD injection in warts, suggesting that this cytokine may play a role in PPD-induced wart clearance. Aktas (6) observed a complete remission among 70% of patients after 1 month of focal treatment with vitamin D; Shah (7) injected MMR vaccines locally into warts and found a complete remission among 72% of patients. Some researchers have found that immunotherapies have other advantages; for example, after a topical injection, warts further away from the injection site and those inside the mucosa that were not injected were found to shrink. They suggest that this may be due to a systemic immune response to local antigens, resulting in a widespread clearance of the HPV virus, which provides an excellent treatment idea for removing warts from problematic areas.

Although there is a wide range of immunotherapies available for treating CA, most are compared with saline placebo, and there is a lack of a direct comparison among the various immunotherapies. Many studies have demonstrated that network meta-analysis can analyze the data from randomized clinical trials to compare the effectiveness of multiple treatments simultaneously without disturbing each treatment. This study aimed to use a network meta-analysis to compare various immunotherapeutic treatments, so as to analyze their effectiveness in treating CA and provide more evidence for our future treatment.

## 2 Method

This study follows the extension statement on preferred reporting items for systematic reviews and meta-analyses (PRISMA) for reporting systematic reviews incorporating network meta-analyses of health care interventions.

### 2.1 Data sources and searches

PubMed, Cochrane Library and Embase were searched from databases from inception to June 1st, 2023 using a computer. All articles on immunotherapies for CA were included. A combination of free and subject words was used when searching the databases. Two researchers independently screened the literatures by title, abstract and full-text review, who discussed to resolve disputes they had encountered.

### 2.2 Selection criteria

The studies were screened for the following inclusion criteria: (1) Study type: randomly-controlled trials (RCTs); (2) Study population: patients diagnosed with CA; (3) Interventions: various immunotherapies, including MMR vaccines, PPD antigens, BCG vaccines, Candida antigens; (4) Outcome indicators: complete clearance rate of immunotherapies.

The studies were also screened for the following exclusion criteria: (1) Duplicate publications; (2) Literatures with only titles and abstracts; (3) Non-English literatures; (4) Academic conference literatures, secondary studies such as reviews or meta-analyses; (5) Literatures with unclear data or information descriptions.

### 2.3 Data extraction and quality assessment

We used standard data extraction forms to extract information, which included authors, year of publication, sample size, adequate sample size, interventions, follow-up time and outcome measures. We used standard criteria (Cochrane risk of bias tool) to assess the inherent risk of bias in trials. Two investigators independently undertook data extraction and quality assessment using a standardized approach. Any disagreement between the two investigators was resolved through consultations with each other.

### 2.4 Data analyses

First of all, we did pair-wise meta-analyses using StataMP 17. The odds ratio (OR) with a confidence interval (CI) of 95% was adopted as a representative measure of dichotomous outcomes. The statistical significance was set as *p* < 0.05. Secondly, a random-effects model network meta-analysis (NMA) was performed to calculate estimates for the efficacy of different interventions against CA. For the outcome, we used a network graph to compare all treatments. Treatment strategies for each outcome were ranked according to the surface under the cumulative ranking curve (SUCRA) probabilities, SUCRA is a method for ranking the strengths and weaknesses of interventions, and a higher SUCRA probability of each simulation indicates a higher chance of being the best treatment regimen. We performed a cluster analysis to find the optimal intervention considering efficacy, and the interventions located in the upper left corner were superior to others.

## 3 Results

### 3.1 Retrieval results of literatures

**Figure 1:**
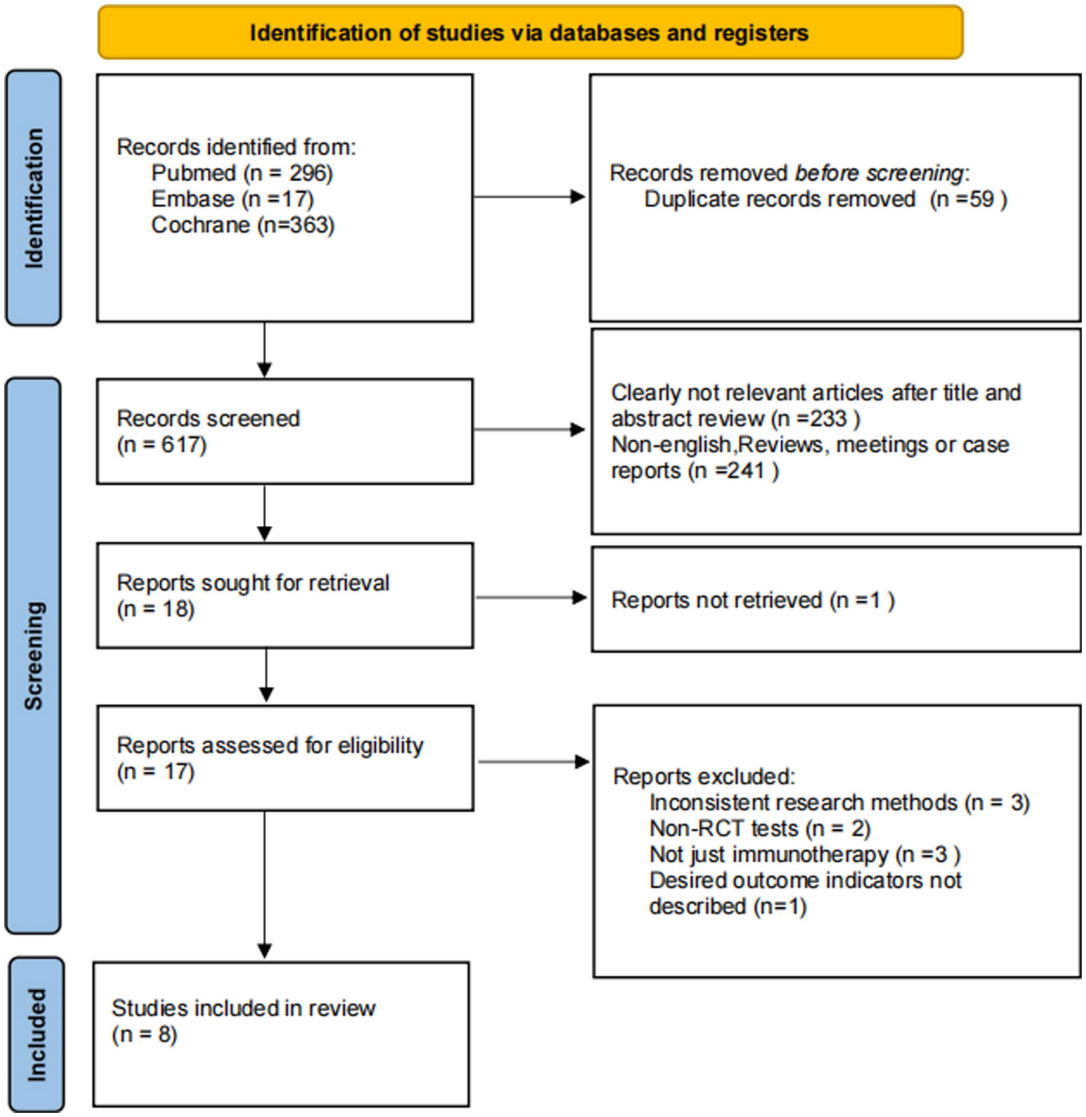
Search flow diagram.

According to the purpose of the study, 676 related articles were initially searched. After screening titles/abstracts and removing duplicate studies, the full text of 18 potentially-eligible studies was obtained. Ultimately, 8 randomized clinical trials were included in the quantitative data synthesis. Figure 1 illustrates the systematic literature search.

### 3.2 Basic characteristics of the included studies

A total of 493 patients were included in the 9 RCTs; the treatments involved were local injection of MMR vaccines, local injection of Candida antigens, local injection of normal saline, local injection of PPD vaccines, systemic injection of PPD vaccines, local injection of vitamin D3 and local injection of BCG vaccines (4, 8–15). The basic information of the literatures is shown in Table 1. Characteristics of included studies; MMR, measle, mumps and rubella vaccines. PPDs, purified protein derivatives. BCG, bacille Calmette-Guerin vaccines. VD3, vitamin D3.

**Table 1:** Characteristics of included studies.

Included studies	Age (years)	Treatment	Sample size	Effective size	Frequency	Treatment duration	Follow-up (months)	Outcome measures
Ahmad 2020 (7)	1–12	MMR	15	11	Once every 2 weeks	Eliminate or up to 5 times	6	complete clearance
		Candida antigen	15	12	Once every 2 weeks	Eliminate or up to 5 times	6	complete clearance
		intralesional saline	10	1	Once every 2 weeks	Eliminate or up to 5 times	6	complete clearance
Noha Z 2022 (3)	21–63	PPD	40	26	Once every 2 weeks	Eliminate up to 4 times	6	complete clearance
		Candida antigen	40	25	Once every 2 weeks	Eliminate up to 4 times	6	complete clearance
Anant 2022 (8)	10–50	MMR	50	31	Once every 2 weeks	Eliminate or up to 3 times	6	complete clearance
		VD3	50	27	Once every 2 weeks	Eliminate or up to 3 times	6	complete clearance
Bayoumy 2011 (10)	20–35	PPD (general)	20	10	Once a weeks	Eliminate or up to 12 times	6	complete clearance
		intralesional saline	20	0	Once a weeks	Eliminate or up to 12 times	6	complete clearance
Nashwa 2023 (11)	18–60	MMR	23	18	Once every 2 weeks	Eliminate or up to 5 times	6	complete clearance
		PPD	23	16	Once every 2 weeks	Eliminate or up to 5 times	6	complete clearance
		PPD (general)	23	13	Once every 2 weeks	Eliminate or up to 5 times	6	complete clearance
Ayman 2020 (12)	39–63	Candida antigen	30	24	Once every 2 weeks	Eliminate or up to 5 times	6	complete clearance
		intralesional saline	20	3	Once every 2 weeks	Eliminate or up to 5 times	6	complete clearance
Bahgat 2005 (13)	30–39	BCG	25	20	Once a week	Eliminate or up to 6 times	6	complete clearance
		intralesional saline	25	0	Once a week	Eliminate or up to 6 times	6	complete clearance
Nofal 2020 (14)	19–45	PPD	32	9	Once every 2 weeks	Eliminate or up to 5 times	6	complete clearance
		Candida antigen	32	12	Once every 2 weeks	Eliminate or up to 5 times	6	complete clearance

### 3.3 Risk of Bias

**Figure 2:**
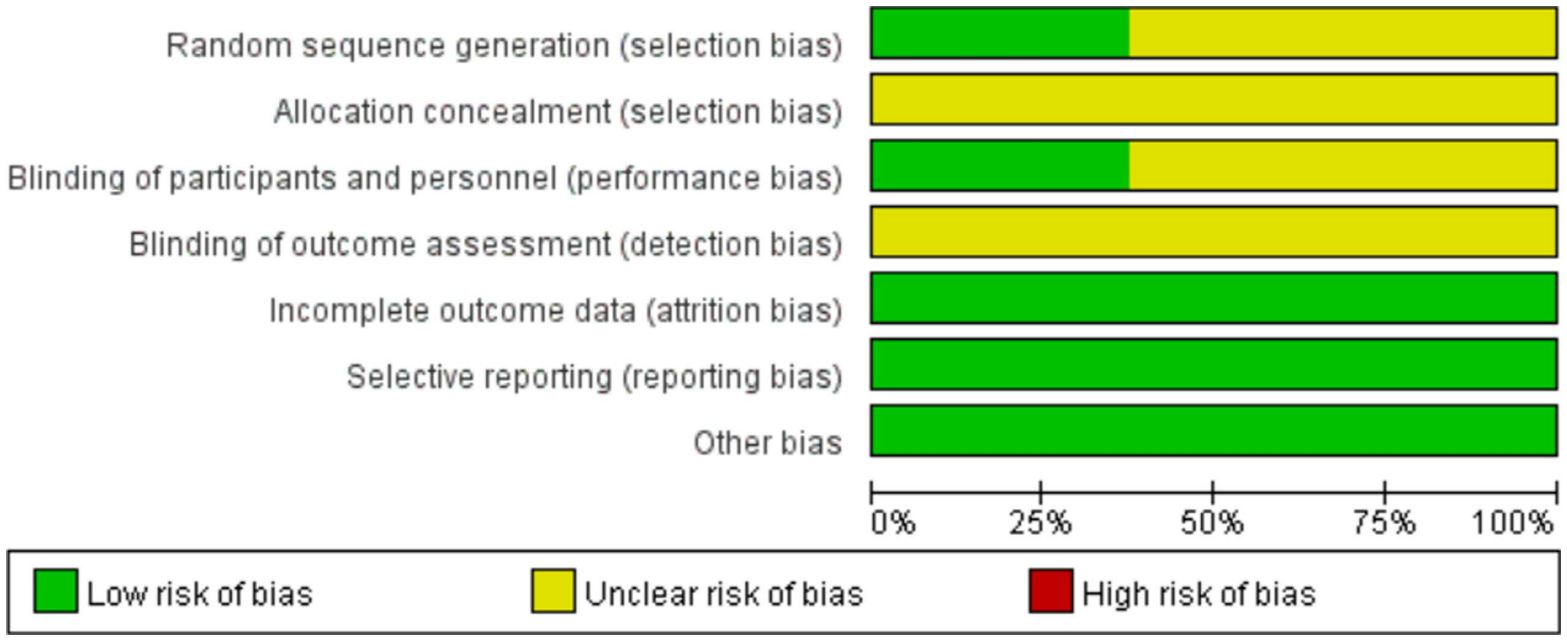
Risk of Bias Summary.

Among the included articles, the random number table was used in 1 of them, random sampling was used in another, coin toss was used in another different from the above 2, and the remaining 5 studies were grouped according to the principle of randomization, but the specific random method was not described; 2 articles were single-blind, 1 was double-blind, and the blindness was not mentioned in the remaining 5 articles; the results of publication biases in studies contributing to outcomes are displayed in Figure 2. The risks of bias were generally low.

### 3.4 Network meta-analysis

#### 3.4.1 Evidence network map

**Figure 3:**
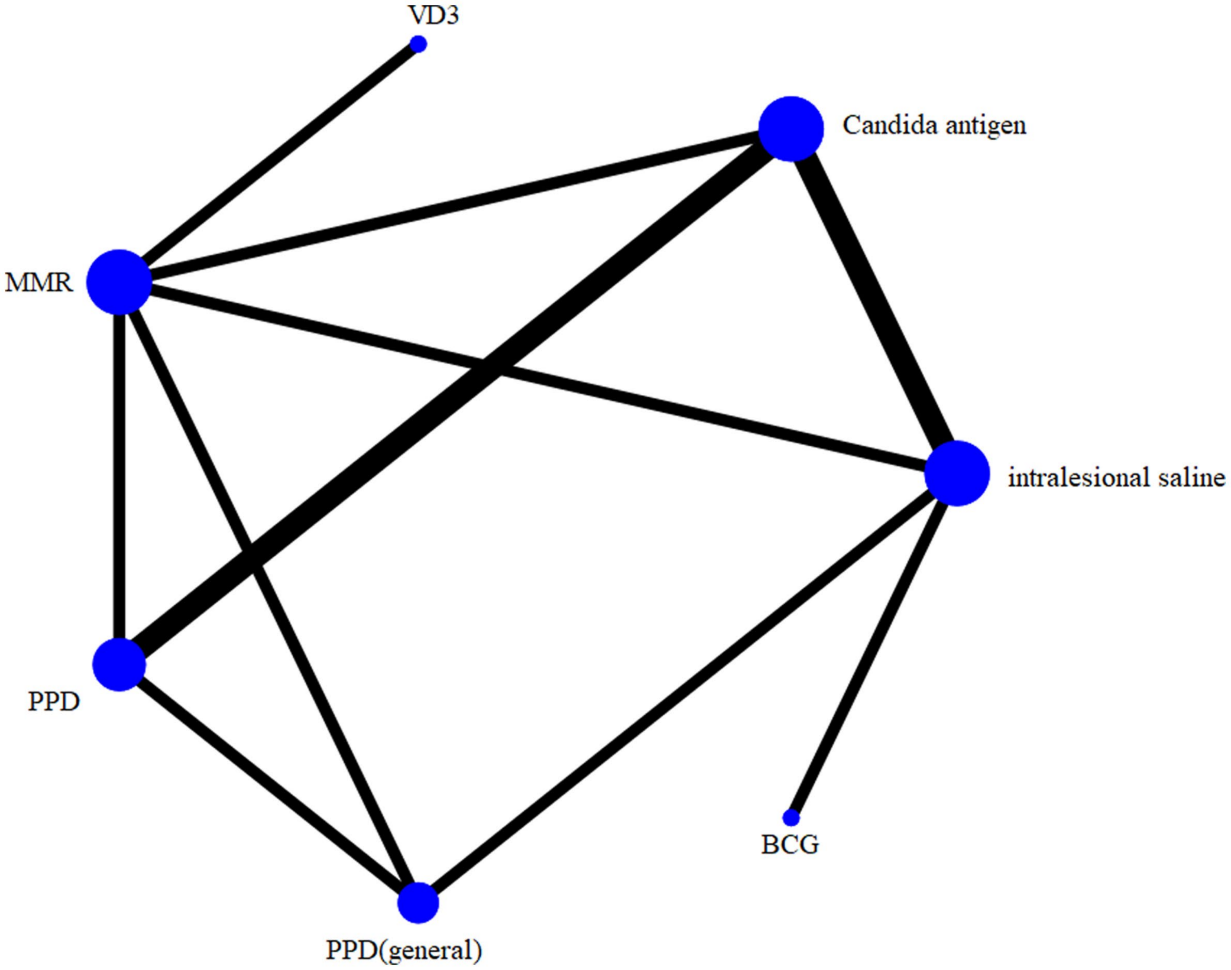
Network geometry.

All the 8 included articles reported a complete clearance, involving 8 treatments, and their sample sizes and direct study evidences are shown in Figure 3. The node size indicates the number of patients randomized to a particular agent. The width of the line indicates the number of trials comparing two treatments. It was found that the samples of Candida antigens ranked the highest in this NMA.

#### 3.4.2 Consistency test

Through an analysis of inconsistency tests on outcome results, it was found that the results of direct and indirect evidences for outcomes were consistent, which should be based on a consistency model (*p* > 0.05).

#### 3.4.3 Results of the network meta-analysis

We used the frequentist method to analyze network meta, with OR as the effect size, and 95% CI inclusion 1 indicated that there was no statistic significance between the two groups. The frequentist method is based on a probabilistic interpretation of frequency, interpreting probability as a stable value of frequency after a large number of repeated trials. Using the frequency method can compare the advantages and disadvantages of treatments by means of a two-by-two comparison. Table 2 shows that all treatment measures have a significant efficacy compared with the standard saline group (BCG OR = 96.00, 95%CI: 10.35–890.58; MMR OR = 29.69, 95%CI: 7.47–118.04; Candida antigens OR = 27.34, 95%CI: 8.64–86.52; PPDs OR = 23.33, 95%CI: 6.75–80.60; VD3 OR = 21.36, 95%CI: 4.34–105.16 and PPDs (general) OR = 13.14, 95%CI: 3.38–51.12).

**Table 2:** Results of network meta-analysis for primary outcomes.

BCG	0.31 (0.02, 4.25)	0.28 (0.02, 3.50)	0.24 (0.02, 3.11)	0.22 (0.01, 3.44)	0.14 (0.01, 1.86)	0.01 (0.00, 0.10)
3.23 (0.24, 44.43)	MMR	0.92 (0.31, 2.72)	0.79 (0.27, 2.26)	0.72 (0.32, 1.60)	0.44 (0.14, 1.40)	0.03 (0.01, 0.13)
3.51 (0.29, 43.11)	1.09 (0.37, 3.21)	Candida antigen	0.85 (0.45, 1.63)	0.78 (0.20, 3.00)	0.48 (0.15, 1.52)	0.04 (0.01, 0.12)
4.11 (0.32, 52.66)	1.27 (0.44, 3.67)	1.17 (0.61, 2.23)	PPD	0.92 (0.24, 3.44)	0.56 (0.19, 1.65)	0.04 (0.01, 0.15)
4.49 (0.29, 69.52)	1.39 (0.63, 3.08)	1.28 (0.33, 4.92)	1.09 (0.29, 4.11)	VD3	0.62 (0.15, 2.50)	0.05 (0.01, 0.23)
7.30 (0.54, 99.22)	2.26 (0.71, 7.15)	2.08 (0.66, 6.58)	1.77 (0.60, 5.21)	1.63 (0.40, 6.60)	PPD (general)	0.08 (0.02, 0.30)
96.00 (10.35, 890.58)	29.69 (7.47, 118.04)	27.34 (8.64, 86.52)	23.33 (6.75, 80.60)	21.36 (4.34, 105.16)	13.14 (3.38, 51.12)	intralesional saline

Table 2 the median of the posterior distribution based on 50,000 simulations was reported as odds ratio (OR), and the corresponding 95% credible intervals (CI) were obtained using the 2.5th and 97.5th percentiles of the posterior distribution, after adjusting for multiple arm trials. Treatments are reported in order of ranking of efficacy.

**Figure 4:**
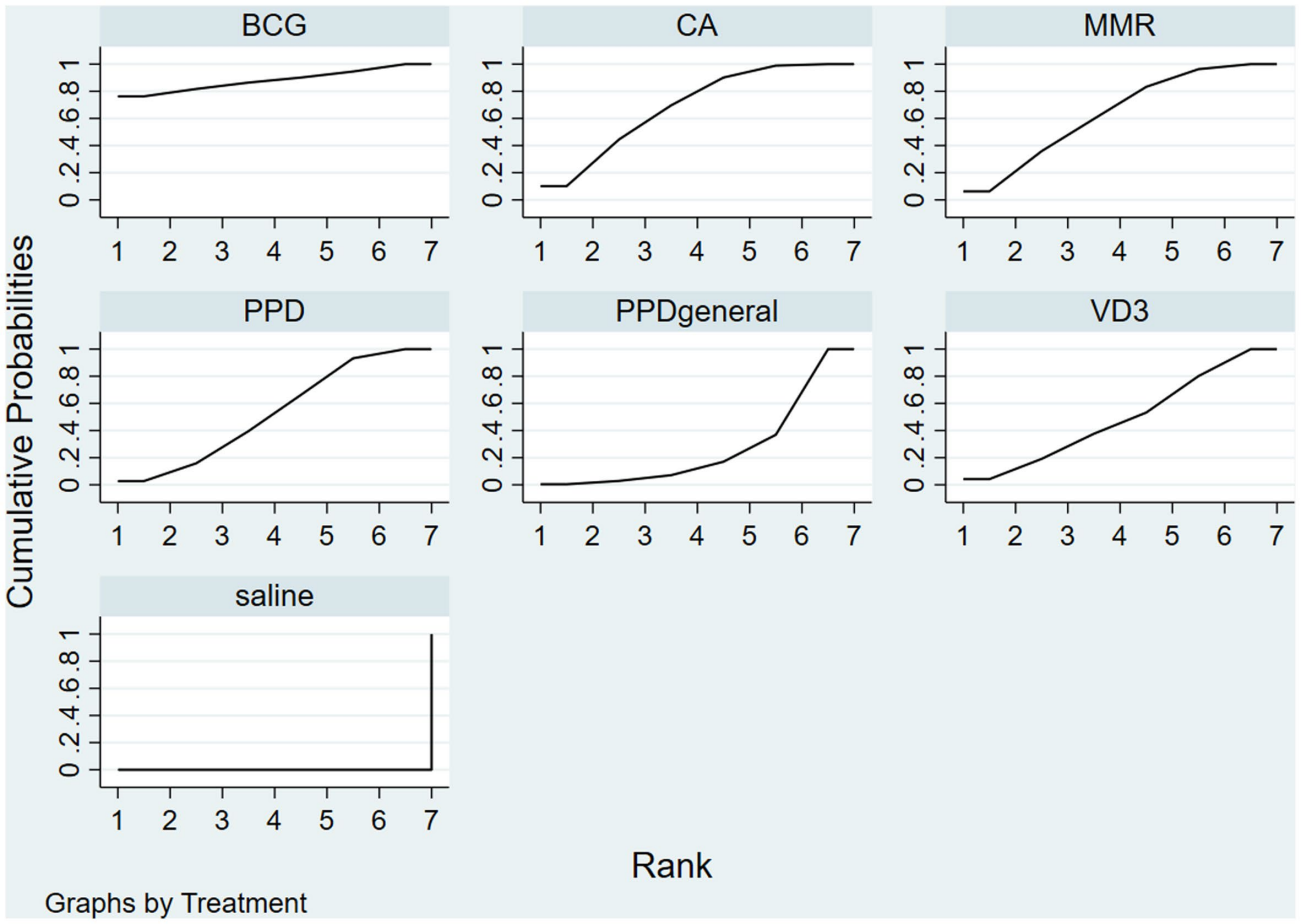
The frequentist method was used to rank for complete clearance probabilistically; efficacy rankings are based on cumulative SUCRA, with larger areas leading to better outcomes. Use this figure as an example, BCG has the largest area under the curve, indicating that it has the best treatment effect.

The frequentist method was used to rank for complete clearance probabilistically; efficacy rankings are based on cumulative SUCRA, with larger areas leading to better outcomes; results show that BCG vaccines 88.2% > MMR vaccines 68.9% > Candida antigens 63.6% > local injection of PPD vaccines 52.9% > vitamin D3 49.0% > systemic injection of PPD vaccines 27.4% > saline 0% in Figure 4.

## 4 Discussion

This is the first NMA to compare the efficacy of all immunotherapies for CA, with 8 RCTs involving 493 patients included in the study. All treatment modalities showed a statistically-significant efficacy compared to placebo. According to the SUCRA results, the BCG vaccines are the most effective for CA among these immunotherapies.

Current medical treatments for CA are mostly topical with stimulant drugs such as imiquimod or cryotherapy to remove warts directly. Although the warts can be removed quickly, the recurrence rate is high, and the results are poor for specific areas such as the intravaginal, intra-urethral and intra-anal areas. The photodynamic therapy is often used to reduce the recurrence rate, but patients often have difficulty tolerating the pains associated with the treatment. Immunotherapies have recently been used to treat warts by injecting an immune extract directly into the warts to achieve their removal. In 1 study, the MMR vaccines were administered topically to 20 children with condyloma warts, 73% of whom showed a complete remission and no recurrence 6 months after the treatment (8). Mild erythema, edema and flu-like symptoms may occur during treatment, which rapidly subside after the administration of NSAIDs, which can provide more excellent relief to patients. Some reduction in warts without immunization has been found in some studies, which may be because local antigens stimulate the body to produce a systemic immune response. Intradermal forearm injections of PPD antigens were used in 1 study to treat 40 pregnant women with CA (11), 74% of whom had a complete wart clearance, reconfirming that antigenic treatment can act on distant warts with a good efficacy. Immunotherapies cannot be used to differentiate between HPV types and their high effectiveness against all types of warts. So topical immunotherapies can be an excellent treatment for areas where traditional lasers and cryotherapies are complicated options, such as the perineal area. Although local injections of immune extracts are commonly used to treat refractory warts, the choice of immunotherapy methods and the dose used are still challenging.

All immunotherapy treatments were more effective than saline, which was statistically significant. Immunotherapies have proven to be effective, which are a new option for our future treatment of refractory CA. In our results, BCG is more effective than immunotherapies. However, it is believed in other literatures that PPD and MMR vaccines may be considered first-line treatments for warts (16). Our analysis may be due to the fact that the literatures do not include randomly-controlled trials of BCG for CA; our literatures show that MMR and PPD treatment is second only to BCG. To some extent, it also confirms the better efficacy of MMR and PPD treatment.

BCG is a live attenuated vaccine made from an artificially cultured suspension of non-toxic bovine *Mycobacterium tuberculosis*, which contains live, attenuated *mycobacterium bovis* and maintains a sufficiently high level of immunogenicity; the immune response it causes is a direct antigen–antibody reaction with a significant local response. However, PPDs are protein derivatives derived from *mycobacterium tuberculosis*, which causes cross-reactivity and a weak local response. This may also explain the superiority of BCG injections (77.8%) over PPD injections (23.8%).

The results show that local PPD injections (45.9%) are more effective than systemic PPD injections (23.8%); likely, PPDs are not immunogenic enough to elicit a strong systemic immune response to clear warts, but only cause a solid local immune response.

Different immunotherapies can be chosen based on patients’ response to the antigens. A small quantity of antigens may be injected intradermally into the forearm of the test hosts for testing before immunotherapies, readings after 48–72 h and the sensitivity observed in the presence of erythema or hard nodules. If a patient shows little or no response to the antigens, the skin test is considered negative, indicating that the patient is likely to be unresponsive to the antigens and the treatment plan should be changed. However, as a novel therapy, immunotherapies have not yet undergone extensive clinical studies; more investigators and subjects need to be involved to further evaluate their efficacy and safety.

Laser and photodynamic therapies have gradually become main methods for reducing photodynamic recurrence. However, the guidelines state that the intralesional antigen immunotherapy has been used for the treatment of warts, and through conventional destructive therapies in combination with immunotherapies, the recurrence of genital warts can be significantly reduced (1). So for stubborn warts or areas where laser treatment is not convenient, we can take immunotherapies or combine them with lasers and drugs for a better treatment of warts.

There are some limitations in this study: (1) In addition, in some of the included trials, whether blinding was used was not mentioned or their randomization and allocation concealment processes were not adequately reported. The quality of the study is not described in great detail, especially in terms of allocation concealment, which is not clear from all the literatures. To reduce biases and make the results more reliable, researchers should follow the CONSORT reporting standards to improve the quality of reporting; (2) This study was focused on the analysis of the efficacy of immunotherapies on CA, which did not include comparative trials of other treatment methods, and immunotherapy approaches were relatively too new to be involved in a larger number of literatures compared to traditional treatments. (3) Due to the lack of data, we did not compare the ranking of the adverse effects or relapse rates among the various immunotherapies, which should also serve as an essential basis for the choice of treatment.

In summary, BCG has an excellent efficacy in the treatment of CA. Perhaps in the future, lasers or cryotherapy can be used to remove warts first, followed by local immunotherapies to achieve better results and reduce recurrence. Due to the quantitative and qualitative limitations of this study, future clinical studies with larger sample sizes, multiple centers and a higher quality will be needed to validate this conclusion.

## Author contributions

XL: Writing – original draft. MQ: Writing – review & editing.
